# Burden of smoking-related stroke in Saudi Arabia: trends from 1990 to 2021

**DOI:** 10.1371/journal.pone.0324039

**Published:** 2025-05-28

**Authors:** Jaber S. Alqahtani, Ibrahim A. AlDraiwiesh, Abdulelah M. Aldhahir, Tope Oyelade

**Affiliations:** 1 Department of Respiratory Care, Prince Sultan Military College of Health Sciences, Dhahran, Saudi Arabia; 2 King Salman Center for Disability Research, Riyadh, Saudi Arabia; 3 Respiratory Therapy Department, Faculty of Applied Medical Sciences, Jazan University, Jazan, Saudi Arabia; 4 Division of Medicine, University College London, London, United Kingdom; 5 School of Medicine, Keele University, Keele, Staffordshire, United Kingdom; Tan Tao University, VIET NAM

## Abstract

**Background:**

Stroke ranks among the top causes of death and disability globally, with smoking being a significant risk factor for its development. This study aims to assess the impact of smoking-related strokes in Saudi Arabia from 1990 to 2021.

**Methods:**

The data was extracted from the Global Burden of Disease (GBD) 2021 database. We assessed the burden of smoking-related stroke by estimating the age-standardized rate (ASR) of years of life lost (YLLs), years lived with disability (YLDs), disability-adjusted life years (DALYs) and deaths related to this disease.

**Results:**

From 1990 to 2021, there was a respective 22.37% and 24.15% absolute decrease in the ASR of DALYs and ASR of YLLs, with average annual percentage changes (AAPCs) of -0.85 (-0.88, -0.83) and -0.92 (-0.94, -0.89) attributable to smoking-related stroke in Saudi Arabia. The ASR of death fell absolutely by 28.48%, with AAPC of -1.08 (-1.11, -1.05) between 1990 and 2021. In contrast, the ASR of YLDs absolutely increased by 5.17% during the same period, with an APCC of 0.15 (0.14, 0.17). Further, the decline in AAPCs of ASR of DALYs (-1.99), ASR of YLLs (-1.79), and ASR of deaths (-1.90) was primarily influenced by the reduction in the female population, p < 0.001, except ASR of YLDs, where the increase in the AAPC was attributed to a rise in the male population, p < 0.001. In 2021, YLLs contributed 93% (157.81/179.02) of total DALYs from smoking-related strokes in Saudi Arabia. Death rates rose in all age groups in 2021, with the most significant increases seen in the younger and middle-aged groups (30–59 years).

**Conclusion:**

While the rates of deaths, DALYs, and YLLs attributable to smoking-related stroke decreased in Saudi Arabia between 1990 and 2021, YLDs significantly increased, mainly in males during the same period. This emphasizes the need for targeted intervention focused mainly on the younger and middle-aged males.

## Introduction

Stroke is a major global concern, imposing a substantial social and economic burden [1]. It accounts for 7.3 million annual deaths and 160.5 million disability-adjusted life years (DALYs) lost globally [2], making stroke the second-leading cause of mortality worldwide [1]. Among the various risk factors associated with stroke, tobacco smoking remains one of the most preventable and persistently prevalent contributors [2,3].

Previous studies reported that active smoking increases the risk of ischemic stroke by 67–85% [4,5]. Moreover, the stroke-related mortality rate due to active smoking increased by 36%, along with a 33.5% increase in DALYs over the past three decades [6]. This can be attributed to the intricate pathophysiological mechanism by which smoking is detrimentally linked with stroke by causing endothelial dysfunction, atherogenesis, and prothrombotic states induced by cigarette toxic components [7]. Further, Ahanger et al, in a recent study showed a significant increase in the incidence of haemorrhagic stroke among smokers and opioid users [8].

Data from the recent Global Burden of Disease (GBD) 2021 reported a global alarming trend regarding smoking-related strokes fuelled by the rising prevalence of smoking aligned with a growing incidence of strokes among the younger population [2]. Despite the current global strides in tobacco control policies to mitigate smoking prevalence across different geographic regions, the burden of smoking-related stroke continues to escalate, particularly in developing nations [9,10]. This highlights the need for a nuanced understanding of the region-specific factors contributing to the health burden caused by smoking-related stroke, specifically where regulatory enforcement of smoking restrictions is often insufficient.

Saudi Arabia has one of the highest smoking prevalence rates across the Eastern Mediterranean region, exceeding 20% among the male population [9]. Although recent advancements in anti-smoking policies were made, including the implementation of the World Health Organization (WHO) Framework Convention on Tobacco Control (FCTC) in 2006 [11], smoking prevalence remains persistent in Saudi Arabia, particularly among the young male population, which exacerbates the incidence and severity of non-communicable diseases, including stroke. Previous studies have established link between smoking and prevalence of stroke. Specifically, in a recent study assessing the risk of acute stroke attributable to smoking in 32 countries from the Middle East and other global regions, Wang et al, reported that smoking is significantly associated with an increase risk of stroke in the study regions [12]. Indeed, smoking remains a major risk factor for all types of strokes in Saudi Arabia [13].

In 2020, stroke incidence is estimated to be around 29 cases for every 100,000 people in Saudi Arabia [14]. However, studies investigating smoking as a risk factor for stroke in Saudi Arabia are lacking despite the rapid increase in smoking prevalence over the years. Moreover, there is an urgent demand for further scrutiny regarding the health, social, and economic burden and trends of smoking-related strokes, which could provide key insights into age-standardized mortality, DALYs, and years lived with disability (YLDs) nationwide.

This study aims to critically examine the burden of smoking-induced strokes in Saudi Arabia over three decades, from 1990 to 2021. By utilizing data from the GBD database, it also seeks to analyze local and global trends to provide evidence-based recommendations for policymakers to strengthen national public health strategies, which ultimately could mitigate the impact of smoking-related strokes.

## Methods

### Data source

This research used data from the Global Burden of Disease (GBD 2021, accessed 01/12/2024) repository via the Global Health Data Exchange (GHDx), a database and website managed by the Institute for Health Metrics and Evaluation (IHME) [15]. Data were retrieved from the GBD database 2021 (released May 2024), encompassing metrics that are vital to quantify the health burden of smoking-related stroke in Saudi Arabia such as disability-adjusted life years (DALYs), Years of life lost (YLLs), years lived with disability (YLDs), and deaths. The GBD 2021 data were collected through an extensive and systematic search of various data sources including large-scale surveys, GBD collaborators, government websites, demographic compendia, and statistical annuals among others. The full details on how this data was sourced specifically for Saudi Arabia can be assessed online at https://ghdx.healthdata.org/gbd-2021/sources. This paper aimed to examine the latest trends in stroke morbidity, measured by (DALYs) and mortality rate attributable to smoking from the year 1990–2021, by sex and age. According to WHO criteria, GBD was defined stroke as a sudden loss of brain function lasting more than 24 hours or resulting in death due to vascular origin and it includes ischemic stroke, intracerebral hemorrhage, and subarachnoid hemorrhage [16,17]. Smoking was defined as any past or current smoke of tobacco [18].

### Estimation framework

The GBD 2021 study protocol and data sources especially how GBD model and determine smoking-related stroke are detailed elsewhere [1,18,19]. We conducted secondary analysis using the GBD 2021 database, which utilized the comparative risk assessment (CRA) framework to compute the burden of disease attributable to smoking risk. CRA framework is anchored on the application of population attributable fractions (PAFs) and based on the ability to measure the extent to which an outcome (stroke) can be prevented if the exposure to a given risk factor (smoking) is reduced to its theoretical minimum risk exposure level (TMREL). According to the GBD 2021 dataset and previous similar studies, the TMREL for smoking was set at 0% prevalence of previous or current tobacco use [20,21]. Thus, the lowest theoretical risk associated with tobacco use is present in a population without any history of use of tobacco. The TMREL for smoking in the GBD dataset was the same across ages. The GBD data have been de-identified and made public; therefore, this study was exempt from review by the institutional ethics board. Also, this study complied with the secondary data access and use policies of the IHME’s terms of use for non-commercial purpose.

### Statistical analysis methods

YLLs were estimated as the product of stroke attributable to smoking deaths, and the remaining life expectancy at the given age was derived from the GBD standard life table. In the same manner, YLDs were computed by multiplying the prevalence rate by the disability weight of a person with the impairment [18]. Thus, the total YLLs and YLDs were computed to determine the DALYs. Generally, the GBD then computes the uncertainty intervals (UIs) of YLL and YLD via the computation of DALYs using Monte Carlo simulations to assess the probability distribution of the input parameters. For mortality estimation, GBD employs the Cause of Death Ensemble model (CODEm), a robust model that corrects for various biases using the combination of various data sources and covariates. Prevalence, incidence, remission and mortality rate are modelled in GBD using the Bayesian mixed-effects meta-regression (DisMod-MR) tool [22].

Initially, we computed the absolute change in percentage of our outcomes with the baseline year (1990) as the point of comparison for each measure. To compare the results with more meaningful comparisons, the age-standardized rates (ASRs) were used to adjust for differences in the population’s age distribution. The GBD age standardization process involves the use of the GBD global standard population as a reference. This standard population is based on the global age distribution and provides a reliable and constant measure of comparison of age-specific rates for any region or period [22]. All analysis included sex stratification to examine the trends between men and women. We reported 95% UI for absolute estimations, with UIs reflecting the 2.5th and 97.5th percentiles of a 1000-draw distribution at each stage. Descriptive and exploratory data analysis were conducted with the help of IBM SPSS statistics version 28 (IBM Corp. Armonk, New York, USA). Further, we used Joinpoint regression to analyze the trends of smoking-related stroke burden measures and mortality rate from 1990 to 2021 with year as the independent variable and measures of burden or mortality as dependent variables. To determine the optimal number of joinpoints and model, a permutation of 4,499 permutations was performed [23]. Using a datapoint of 31, equivalent to the years from 1990 to 2021, the Grid search method was used to determine the range of joinpoints between 0 and 5. Annual percentage change (APC) with 95% confidence interval was computed for each joinpoint. For the entire period between 1990 and 2021, weighted average annual percent changes (AAPC) was also computed along with their 95% CI for all variables. In summary, the AAPC reflects the overall percentage change in trend between the initial period (1990) and last year of record (2021) whereby AAPC is equivalent to APC if joinpoint = 1. For the Joinpoint analysis, we used the Joinpoint Regression Program (version 5·2·0, June 2024; Statistical Methodology and Applications Branch, National Cancer Institute) with an overall p value < 0.05 considered to reflect statistically significant change (increase or decrease) in YLLs, YLDs, DALYs, or Mortality rates. Where p value is not presented, lack of overlap confidence or uncertainty intervals was used to interpret statistical significance. All analysis was stratified based on sex.

## Results

The DALYs ASR of stroke attributable to smoking decreased absolutely by 22.37% from 1990 to 2021. Similarly, the ASR of YLLs declined from 207.44 per 100,000 in 1990 to 157.81 per 100,000 in 2021, reflecting an absolute reduction of 24.15%. The ASR of deaths decreased from 7.69 per 100,000 in 1990 to 5.53 per 100,000 in 2021, resulting in a 28.09% absolute reduction. Au contraire, the YLDs ASR increased from 11.61 per 100,000 in 1990 to 12.20 per 100,000 in 2021, reflecting a growth of 5.17%. The total number of stroke deaths attributed to smoking increased from 480 in 1990 to 1408 in 2021, reflecting a rise of 193.33% as an absolute change. The joinpoint analysis shows findings similar to the absolute percentage change above. Specifically, there was a significant decrease in ASR of DALYs, ASR of YLLs, and ASR of deaths between 1990 and 2021, with the highest decrease observed in females. Also, only an increase was observed in the ASR of YLDs with AAPC of 0.15% (95% CI = 0.14 to 0.17), p < 0.001) (Table 1).

**Table 1:** Trends in the burden of stroke attributed to smoking.

Measure	1990 (95% UI)	2021 (95% UI)	AAPC (95% CI)	P value
**Age-standardized DALYs Rate per 100,000 Population**				
Male	331.61 (236.70-458.50)	258.90 (195.30-338.30)	-0.82 (-0.88, -0.80)	< 0.001
Female	54.55 (34.95- 79.63)	32.12 (20.36-46.71)	-1.99 (-1.42, -11.62)	< 0.001
Both	219.06 (156.45-299.91)	170.02 (127.89-220.52)	-0.85 (-0.88, -0.83)	< 0.001
**Age-standardized YLDs Rate per 100,000 Population**				
Male	17.43 (12.41-23.68)	18.48 (12.98-24.87)	0.18 (0.16-0.20)	< 0.001
Female	2.62 (1.69-3.92)	2.39 (1.50-3.59)	-0.31 (-0.33, -0.30)	< 0.001
Both	11.61 (8.22-15.76)	12.20 (8.51-16.43)	0.15 (0.14, 0.17)	< 0.001
**Age-standardized YLLs Rate per 100,000 Population**				
Male	314.17 (220.17- 438.48)	240.46 (179.37- 317.33)	-0.89 (-0.92, -0.86)	< 0.001
Female	51.92 (33.25- 76.90)	29.72 (18.54- 43.60)	-1.79 (-1.84, -1.74)	< 0.001
Both	207.44 (146.74- 286.53)	157.81 (117.13- 205.68)	-0.92 (-0.94, -0.89)	< 0.001
**Age-standardized Death Rate per 100,000 Population**				
Male	12.10 (8.44- 17.00)	8.50 (6.27- 11.37)	-1.16 (-1.18, -1.13)	< 0.001
Female	1.85 (1.15- 2.78)	1.02 (0.64- 1.53)	-1.90 (-1.96, -1.84)	< 0.001
Both	7.69 (5.42- 10.73)	5.53 (4.09- 7.35)	-1.08 (-1.11, -1.05)	< 0.001

*AAPC: Average Annual Percent Change; YLD: years lived with disability; YLL: years of life lost, DALY: Disability-adjusted life years, UI: uncertainty Interval; CI: Confidence Interval

### Morbidity caused by stroke attributed to smoking in Saudi Arabia

#### Age-standardized disability-adjusted life years (DALYs).

**Fig 1:**
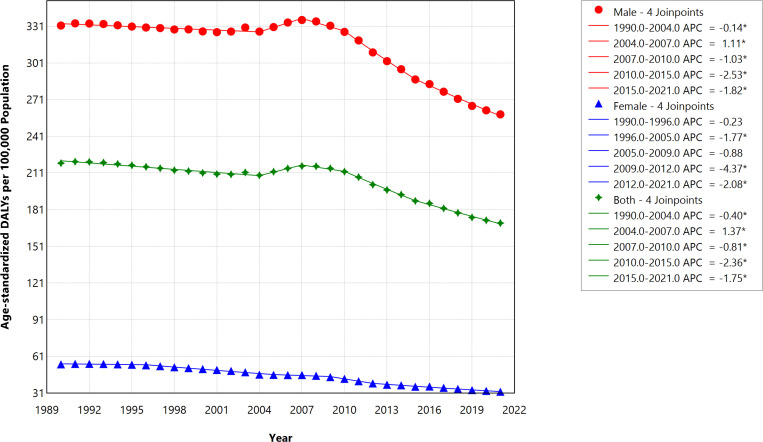
Trends in age-standardized rate of DALYs per 100,000 with caused by smoking-related strokes in Saudi Arabia during 1990–2021, stratified by gender.

In 2021, the ASR of DALYs per 100,000 females decreased to 32.13 (95% UI, 20.3–46.7) from 54.55 (95% UI, 34.96–79.63) in 1990, a 41.10% absolute drop. The ASR of DALYs per 100,000 males reduced from 331.6 (95% UI, 236.7–458.5) in 1990 to 258.9 (95% UI, 195.3–338.3) in 2021, representing a 21.92% absolute reduction. Fig 1 depicts the trends of ASR of DALYs per 100,000 caused by smoking-related strokes in Saudi Arabia from 1990 to 2021, stratified by gender. The annual percentage change (APC) and AAPC based on the joinpoint analysis show that the decrease in the ASR of DALYs is mainly driven by the female population (Female = -1.99, 95% CI = -1.42, -11.62 vs. Male = -0.82 (-0.88, -0.80), p < 0.001), (Table 1 and Fig 1).

#### Years lived with disability (YLDs).

**Fig 2:**
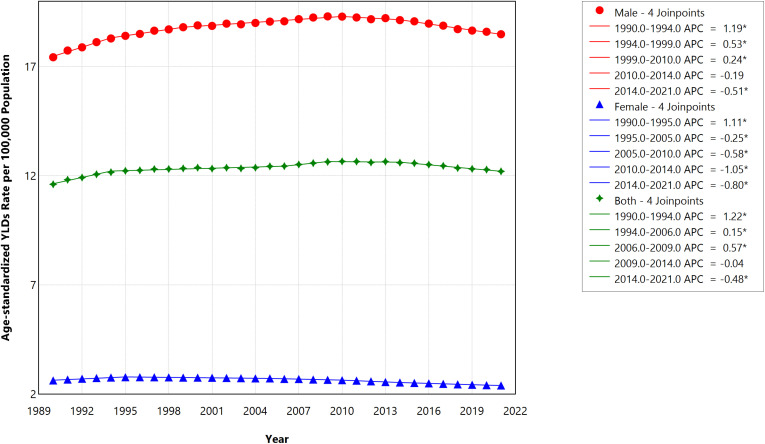
Trends in the age-standardized rate of YLDs per 100,000 caused by smoking-related strokes in Saudi Arabia during 1990–2021, stratified by gender.

In 2021, the ASR of YLDs per 100,000 in males increased to 18.4 (95% UI, 12.9–24.8) from 17.4 (95% UI, 12.4–23.6) in 1990, reflecting a 5.75% absolute increase. The ASR of YLDs per 100,000 females decreased from 2.6 (95% UI, 1.7–3.9) in 1990 to 2.4 (95% UI, 1.5–3.6) in 2021, indicating a 7.69% absolute reduction. Fig 2 illustrates the trends in ASR of YLDs per 100,000 population attributed to smoking-related strokes in Saudi Arabia over the period from 1990 to 2021, categorized by gender. Over the study period, joinpoint analysis shows that the increase in the AAPC of ASR of YLDs in both sexes (0.15, 95%CI = 0.14 to 0.17, p < 0.001) was mainly driven by the increase observed in the male population (Male = 0.18, 95% CI = 0.16 to 0.20 vs Female = -0.31 95% CI = -0.33 to -0.30) (Tables 1 and 2). In 2021, YLDs contributed to around 7% (12.20/179.02) of total DALYs caused by stroke attributed to smoking in Saudi Arabia.

#### Years of life lost (YLLs).

**Fig 3:**
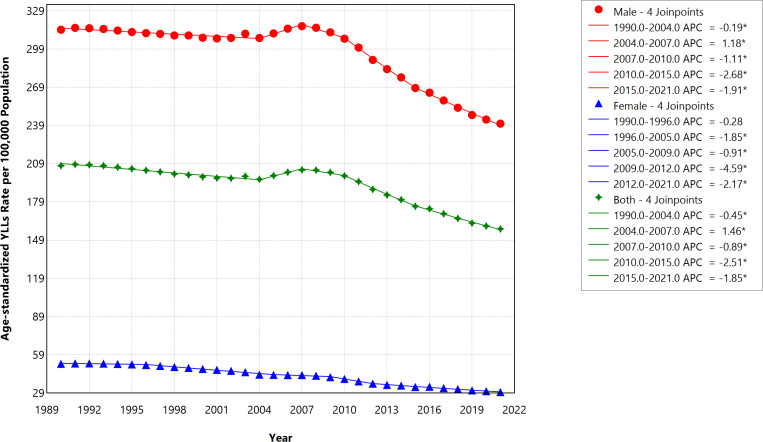
Trends in the age-standardized rate of YLLs per 100,000 caused by smoking-related strokes in Saudi Arabia during 1990–2021, stratified by gender.

Between 1990 and 2021, the ASR of YLLs per 100,000 population attributed to smoking-related strokes in Saudi Arabia showed significant reductions across genders. Among males, the rate decreased absolutely by 23.46%, from 314.1 (95% UI: 220.1–438.4) in 1990 to 240.4 (95% UI: 179.3–317.3) in 2021. For females, the rate declined absolutely by 42.77%, from 51.9 (95% UI: 33.2–76.9) in 1990 to 29.7 (95% UI: 18.5–43.6) in 2021. In 2021, YLLs accounted for approximately 93% (157.81/179.02) of the total DALYs resulting from stroke attributed to smoking in Saudi Arabia. Fig 3 highlights these trends, presenting the ASR of YLLs rates per 100,000 population over this period, categorized by gender. Like DALYs, the decrease in percentage change in ASR of YLLs was significantly driven by the female population across almost all joinpoints (except 2007–2010) with AAPC of -1.79 (95% CI = -1.84 to -1.74, p < 0.001) in females vs. -0.89 (95% CI = -0.92 to -0.86, p < 0.001) (Table 1 and Fig 3)

### Mortality caused by stroke attributed to smoking in Saudi Arabia

**Fig 4:**
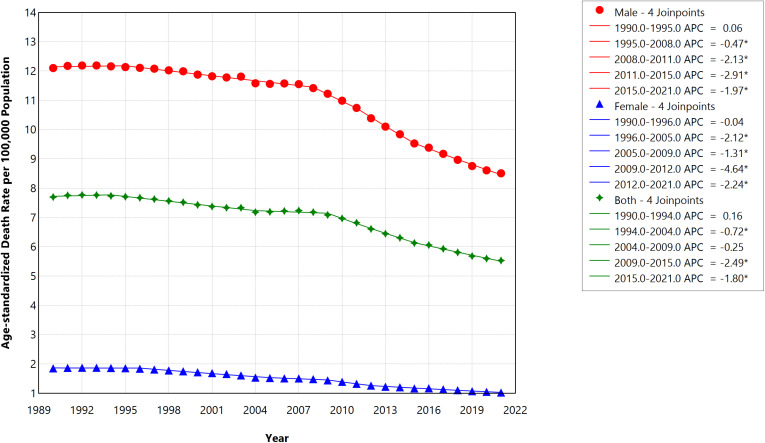
Trends in age-standardized rate of deaths per 100,000 caused by smoking-related strokes in Saudi Arabia during 1990–2021, stratified by gender.

**Fig 5:**
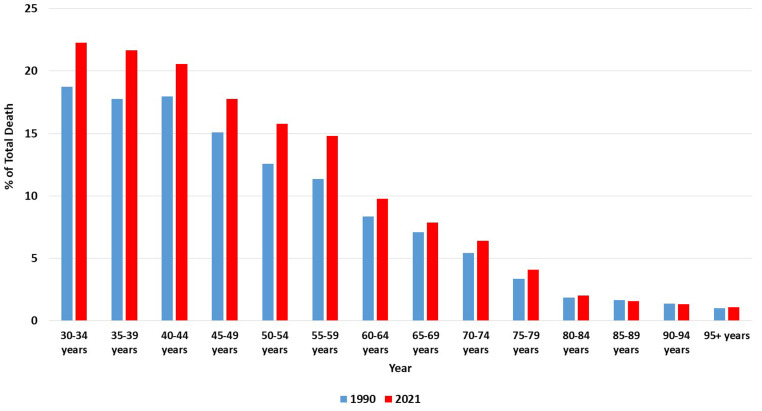
Percentage of total deaths caused by smoking-related strokes in Saudi Arabia (1990 and 2021), stratified by age.

By 2021, the ASR of deaths for males in Saudi Arabia had decreased to 8.5 per 100,000 (95% UI, 6.2–11.3) from 12.1 (95% UI, 8.4–17.0) in 1990, reflecting a 29.75% absolute decline. For females, the ASR of deaths per 100,000 dropped from 1.8 (UI, 1.1–2.7) in 1990 to 1.0 (UI, 0.6–1.5) in 2021, marking a 44.44% absolute reduction. Fig 4 illustrates the ASR of deaths due to smoking-related strokes per 100,000 population in Saudi Arabia from 1990 to 2021, stratified by gender. The trends in ASR of deaths similarly show a decrease between 1990 and 2021, mainly driven by the female population (especially from 2009 onwards) with AAPC of -1.90 (95%CI = -1.96 to -1.84) in females vs -1.16 (95%CI = -1.18 to -1.13) in males (Table 1 and Fig 4). Fig 5 shows that, although death percentages are rising across most age categories, the most significant increases are seen in the younger and middle-aged groups (30–59 years), with lower decreases especially seen in those ≥60 years old.

## Discussion

In this study of smoking-related stroke trends and effects in Saudi Arabia based on the GBD 2021, we found a respective 22% and 24% overall absolute decrease in the ASR of DALYs and YLLs across all genders between 1990 and 2021. Although YLDs increased by 5%, the rate of mortality attributable to smoking-related stroke decreased by 28% between 1990 and 2021. Our analysis shows a steady decrease in the ASR of DALYs across both genders until 2004 before a sharp rise among males peaked in 2007 before steadily falling (APC = 1.11, p value <0.001).

Essentially, a steeper decline was shown in the DALYs rate from 2007 in both genders, representing a turning point in this trend of smoking-related stroke in Saudi Arabia. Like the DALYs rate in Saudi Arabia, the trends in the YLLs between 1990 and 2021 show similar dynamics. Specifically, we observed a steady decrease in both genders while the fall in ASR of YLLs was reversed in 2004, increasing steadily until 2007 (APC = 1.46, p value <0.001) before sharply decreasing until 2021. Our result also shows a steady decline between 1990 and 2021 in the ASR of deaths per 100,000 from smoking-related stroke. Interestingly, a sharp reduction in the ASR of deaths was also observed between 2009 and 2015 (APC = -2.49, p value <0.001) among both genders (Fig 4). These trends highlight that 2003 and 2004 represent an important epidemiological juncture for burden of smoking-related stroke, especially among male smokers in Saudi Arabia.

The sharp decline in the ASR of DALYs, deaths, as well as YLLs linked with smoking-related stroke in 2004 is interesting, especially since the trends in smoking rate have been shown to increase around the same period. For instance, according to the 2013 Saudi Health Information survey, smoking prevalence increased from 12.2% to 15.3% between 2005 and 2013 [24]. Possible factors that could explain this burden reduction despite increased smoking prevalence include improvement in care for stroke patients in Saudi, reduction in other related risk factors for stroke, and changes in GBD modeling methodology over time.

There are various hypotheses for the sharp reduction in the ASR of DALYs, YLLs, and deaths associated with smoking-related stroke in Saudi Arabia from 2007 onwards. For instance, the government of Saudi Arabia became a signatory to the 2003 World Health Organization (WHO) Framework Convention on Tobacco Control (FCTC), developed in response to the global tobacco epidemic and was aimed at reducing the demand for tobacco through financial and non-financial approaches [25]. After ratifying the FCTC treaty, the government of Saudi Arabia immediately initiated a series of national approaches, including legislations and regulations, a 100% increase in tobacco tax, a public awareness campaign aimed at educating and warning people of the harms of smoking, as well as strengthening of the health services to bolster smoking cessation and quitting [11,24,26].

Another risk factor for smoking-related stroke is second-hand smoking [27–30]. To reduce the prevalence and associated effects of second-hand smoke, the Saudi government implemented various regulations, especially around 2007, to create smoke-free zones through the prohibition of smoking in indoor workplaces, public transport, and public areas such as government, cultural, as well as in educational facilities [31]. Obesity is another modifiable risk factor that may elevate the risk of smoking-related stroke and adversely affect overall health outcomes [32,33]. Indeed, other initiatives that may explain the continued decline in the effect of smoking-related stroke on quality of life in Saudi Arabia include the National Campaign against Overweight and Obesity initiated by the Ministry of Health in 2012. This initiative was created to educate and inform the Saudi public about the health effects of overweight and obesity and provide practical advice on lifestyle changes that could enhance weight loss, such as physical exercise and improved nutrition [34]. The launch of Saudi Vision 2030 in 2016 could be a contributing factor to the ongoing decline in trends of ASR of DALYs, YLLs, and deaths until 2021, which focuses on increasing health access, quality as well as awareness to improve the health of Saudi Arabians through public health campaigns and targeted messaging [35]. Albeit no studies have documented the direct effect of some of these initiatives on the health effects of smoking-related stroke, the dynamics shown in this study coincide with the timelines of introduction of the described policies.

Although the ASRs of fatalities, YLLs, and DALYs have declined in 2021 relative to 1990, an increasing number of Saudi Arabians are experiencing disabilities due to smoking-related strokes, as shown by the increase in ASR of YLDs (AAPC of 0.15, p-value <0.0001). This was mainly driven by the increase observed in the male population (Male AAPC = 0.18, 95% CI = 0.16 to 0.20 vs Female AAPC = -0.31 95% CI = -0.33 to -0.30).

The stratification by age range showed that between 1990 and 2021, the percentage of deaths from smoking-related stroke is significantly concentrated among those between the ages of 30 and 59 years. Indeed, previous studies have reported this age range as having the highest prevalence of smoking in Saudi Arabia. While various factors have been proposed to be drivers of smoking among these age groups, including stress, entertainment, bonding with family, peer pressure, etc. [36,37], future studies can investigate how much of these subpopulations end up with stroke. Importantly, these findings highlight the need to refocus future initiatives and campaigns toward these age ranges to effectively increase smoking cessation and reduce smoking-related stroke in the population.

COVID-19 is associated with an increased risk of developing stroke [38] and raises the probability of poorer outcomes in patients with stroke [39] and other chronic diseases [40–42]. Also, the effect of the COVID-19 outbreak in 2019 on the trends of various infectious and non-infectious as well as global life expectancy is well documented in the literature [43]. For instance, in a systematic review and meta-analysis by Alqahtani et al., admissions for COPD exacerbations fell by 50% during the COVID-19 pandemic [44]. Similarly, the global epidemiology, clinical management, and outcomes of various infectious diseases, including influenza virus and HIV, were disrupted during the COVID-19 pandemic [45–47]. Thus, it may be expected that the trends in smoking-related stroke and associated health outcomes in Saudi Arabia would have likely increased. However, we found no changes in the trend of the health effects of smoking-related stroke in this study. Indeed, this may be owing to inconsistent results from studies on whether COVID-19 changed Saudi Arabian smoking habits [48–50].

There are some limitations associated with this study. First, due to the lack of granularity of the GBD dataset, it was impossible to assess the effect of smoking type and intensity on the development of stroke as well as the health outcomes. Specifically, previous studies have shown a dose-dependent relationship between smoking and the risk of stroke, with risk increasing relative to increasing intensity and/or duration [51,52]. However, the GBD does not account for this variation in risk in the estimation of CRA and DALY, which could potentially introduce bias. Second, there is generally a regional difference in smoking behaviors in Saudi Arabia, which may likely feed into regional differences in trends of DALYs, YLDs, YLLs, and deaths rates. However, we could not assess this since the GBD dataset does not include prevalence of different regions and provinces. Indeed, there are various limitations particularly inherent to the GBD dataset that could potentially affect the findings of this study. For instance, issues related to low representation, especially of rural and low-resourced regions of the world (including some regions in Saudi Arabia), under-reporting of risk factors, and the variability in case definition (which may result in misdiagnosis and misclassification) may reduce the reliability of the dataset and the findings herein [53]. Other limitations of GBD include using modelled estimates instead of primary data, the ecological approach limiting causal inference, and methodological changes over time may have influenced trends. Finally, although the 95% UIs of some of the absolute rates did overlap (Table 1) and can be interpreted as showing no statistically significant difference across the years, joinpoint analysis and computed AAPCs established that there are true trends and changes are significant.

Despite these limitations, the GBD dataset represents the largest multinational, multi-year dataset on the health effects of various diseases, injuries, and risk factors sufficiently stratified based on gender, age, and other valuable factors and provides a unique opportunity to understand the epidemiological dynamics of various diseases. Also, while various factors, including socioeconomic status, hypertension or other comorbidities, may have confounding and mediation effects on the estimation of GBD measures, GBD analysis controls for these effects, such as using the CRA framework and complex statistical methods. Indeed, this analysis is the first of its kind, providing insights that may allow both the Saudi government and other stakeholders to understand the health effects of smoking-related stroke over the last 30 years so that resources can be refocused effectively and efficiently.

This research presents several significant future implications. Our data show the dynamics of smoking-related stroke burden from 1990 to 2021, revealing differences between males and females. The total reduction in health burden since 2009 underscores the importance of the national implementation of the WHO FCTC framework in 2003 and the efficacy of subsequent policy reforms. This trend promotes ongoing efforts to maintain current regulations and underscores the need for supporting research in this domain to mitigate smoking-related strokes in Saudi Arabia further. Additionally, targeted awareness is necessary for smokers to emphasize the associated risks and promote smoking cessation. It is required to enhance the incorporation of stroke prevention into standard practice by screening for smoking-related illnesses, including stroke, to facilitate early intervention. It is also necessary to create national registries for stroke and smoking-related disorders to collect more data that would inform future health policy decisions. This study’s results indicate that the burden of smoking-related risk is disproportionately greater among males, which is consistent with previous regional studies [54–56]. This underscores the need to customize campaigns, educational efforts, and therapeutic advice for each sex. By adopting the aforementioned comprehensive strategy and implementing continuous review and modification to address the evolving dynamics of smoking patterns and stroke incidence, Saudi Arabia may achieve substantial progress in alleviating the burden of smoking-related strokes.

## Conclusion

Even though the rates of deaths, DALYs, and YLLs from smoking-related stroke were reduced in Saudi Arabia in the period 1990–2021, YLDs increased mainly among men. This shows that there is still a need for intensive preventive measures, especially for young and middle-aged men.
